# Mixture models for gene expression experiments with two species

**DOI:** 10.1186/1479-7364-8-12

**Published:** 2014-08-01

**Authors:** Yuhua Su, Lei Zhu, Alan Menius, Jason Osborne

**Affiliations:** 1 Dr. Su’s Statistics & Department of Human Nutrition, Food, and Animal Sciences, University of Hawaii at Manoa, Honolulu, HI 96822, USA; 2 Biomarker and Predictive Analytics, GlaxoSmithKline, 5 Moore Drive, Research Triangle Park, NC 27709, USA; 3 Department of Statistics, North Carolina State University, Raleigh, NC 27695, USA

**Keywords:** Orthology, Drug development, Drug response prediction, Type II diabetes

## Abstract

Cross-species research in drug development is novel and challenging. A bivariate mixture model utilizing information across two species was proposed to solve the fundamental problem of identifying differentially expressed genes in microarray experiments in order to potentially improve the understanding of translation between preclinical and clinical studies for drug development. The proposed approach models the joint distribution of treatment effects estimated from independent linear models. The mixture model posits up to nine components, four of which include groups in which genes are differentially expressed in both species. A comprehensive simulation to evaluate the model performance and one application on a real world data set, a mouse and human type II diabetes experiment, suggest that the proposed model, though highly structured, can handle various configurations of differential gene expression and is practically useful on identifying differentially expressed genes, especially when the magnitude of differential expression due to different treatment intervention is weak. In the mouse and human application, the proposed mixture model was able to eliminate unimportant genes and identify a list of genes that were differentially expressed in both species and could be potential gene targets for drug development.

## Introduction

### Background

Pharmaceutical medicine is an industry with huge up-front investment for rewards that may or may not come years later. A complete drug development process, including drug discovery, preclinical research (on animals) and clinical trials (on humans), is lengthy, expensive, and risky. Determined by the US Food and Drug Administration (FDA) [1], the average total cost per drug development is about $1.9 billion. The typical development time is 10 to 15 years. The overall attrition rate of a drug compound from first-in-man to registration is approximately 80%–90% [2,3].

FDA [1] calls the preclinical and clinical research together as the ‘critical path’ development phase, where most investment required for a successful drug launch occurs. Currently, this development phase is inherently inefficient. The goal of preclinical research is to assess how a drug is absorbed, distributed, metabolized, and excreted in animals, and to use the findings to determine potential human outcomes before starting clinical trials. Yet the rate of success after a drug candidate enters Phase I is undesirably low. As mentioned in FDA [1] and Kola and Landis [3], animal models with poor clinical relevance may be accountable for this perplexity. Hence, improving translation between two species to increase the predictive power of animal models to human studies is of tremendous value to drug discovery and development.

### Homology and multiple species gene expression analysis in drug development

Microarrays are tools for gene expression analysis and can be potentially useful for investigating the mechanism of drug activities that translates across species. The utility of microarray information in the drug development process is reviewed by Braxton and Bedilion [4] who embraced the idea that gene expression analysis can be a surrogate marker for the interaction between compounds and cells and should yield information about efficacy. Debouck and Goodfellow [5] believed that microarrays can be used to generate clues to patterns of gene function that can help improve the efficiency of drug development.

As stated before, one key challenge of drug development is to successfully translate the results of preclinical findings in animal models to human beings in the clinic. Pre-clinical experiments assume that the effect of the drug tested on animals is comparable to that on humans, which can only be true if a functional equivalent of the human drug target exists in the experimental species. Orthology [6-8] is a strong indication of functional conservation and therefore provides the best functional annotation of experimentally undetermined genes across species. Holbrook and Sanseau [9] remarked that the use of orthologs has the potential to improve the understanding of biological differences between species (animals and humans).

Many of the successful applications of cross-species microarray gene expression analysis involve orthology [10-13]. Additionally, over the past decade, researchers have tried to use orthology and gene expression data to do cross-species comparison in order to understand how genes interact to perform particular biological processes [14,15]. These studies support the idea that orthologs could be a useful tool for researchers to link experiments between species in drug development. Note that orthologous relationships can be one-to-one, one-to-many, or many-to-many [8].

The rest of the paper is structured as follows: Section ‘Joint modeling across species’ describes the proposed bivariate mixture model across species. Section ‘Simulation’ describes a simulation study undertaken to investigate the effects of different experimental designs on the power to detect important genes and on misclassification rates. Section ‘Application: the mouse and human type II diabetes experiment’ illustrates the methodology using an application to data collected in a mouse/human experiment. Section ‘Concluding remarks’ concludes.

## Joint modeling across species

Let $X_{a_{\text{ij}}}$ and $X_{h_{\text{il}}}$ denote gene expression measurements from the *i*th orthologous gene pair for the *j*th animal and the *l*th human. The following independent linear models describe the association between gene expression and treatment:

(1) $$\begin{array}{c} X_{a_{\text{ij}}}=\beta_{0a_{i}}+\beta_{1a_{i}}T_{a_{j}}+e_{a_{\text{ij}}}, \end{array}$$

(2) $$\begin{array}{c} X_{h_{\text{il}}}=\beta_{0h_{i}}+\beta_{1h_{i}}T_{h_{l}}+e_{h_{\text{il}}}, \end{array}$$

where $T_{a_{j}}$ and $T_{h_{l}}$ are {0,1} treatment indicators, and $e_{a_{\text{ij}}}$ and $e_{h_{\text{il}}}$ are independent $N(0,\sigma_{a}^{2})$ and $N(0,\sigma_{h}^{2})$ random variables. $\sigma_{a}^{2}$ and $\sigma_{h}^{2}$ are variances for $e_{a_{\text{ij}}}$ and $e_{h_{\text{il}}}$, respectively. In drug development, the animal research and human experiments are conducted independently - one’s results do not affect the other’s. However, the treatment effects are expected to have some kind of association between the two species. This results in our choice of using two independent models for the two species to capture the effects of treatment on gene expression.

### A nine-component bivariate mixture model for two species experiments

$\beta_{1a_{i}}$ and $\beta_{1h_{i}}$ quantify the differential expression of the *i*th orthologous animal and human genes due to a treatment intervention. A given gene can be classified as non-differentially expressed (NDE) - showing no signs of treatment effects, positively differentially expressed (pDE) - showing positive treatment effects, or negatively differentially expressed (nDE) - showing negative treatment effects. Therefore, for a human and animal gene pair, there are nine possibilities for categorizing this pair of genes. Further, dependency is assumed between differentially expressed orthologs, i.e., existence of association posited only for ${\left(\beta_{1a_{i}},\beta_{1h_{i}}\right)}^{T}$ in categories (1, 2, 3, 4) and zero correlation presumed for ${\left(\beta_{1a_{i}},\beta_{1h_{i}}\right)}^{T}$ in categories (0, 5, 6, 7, 8). Table 1 illustrates the nine possible categories of ${\left(\beta_{1a_{i}},\beta_{1h_{i}}\right)}^{T}$. ${\left(\mu_{\beta_{1a_{i}}},\mu_{\beta_{1h_{i}}}\right)}^{T}$ is the vector of population means of ${\left(\beta_{1a_{i}},\beta_{1h_{i}}\right)}^{T}$ under each category.

**Table 1:** **Possible categories of**${\left(\beta_{1a_{i}},\beta_{1h_{i}}\right)}^{T}$

**Category**	$\left(\beta_{1a_{i}},\beta_{1h_{i}}\right)$	$\left(\mu_{\beta_{1a_{i}}},\mu_{\beta_{1h_{i}}}\right)$	**Corr**$\left(\beta_{1a_{i}},\beta_{1h_{i}}\right)$
0	(NDE,NDE)	(0,0)	0
1	(pDE,pDE)	(+,+)	*ρ*_1_
2	(nDE,nDE)	(-,-)	*ρ*_2_
3	(pDE,nDE)	(+,-)	*ρ*_3_
4	(nDE,pDE)	(-,+)	*ρ*_4_
5	(NDE,pDE)	(0,+)	0
6	(NDE,nDE)	(0,-)	0
7	(pDE,NDE)	(+,0)	0
8	(nDE,NDE)	(-,0)	0

In consequence of these possible patterns of ${\left(\beta_{1a_{i}},\beta_{1h_{i}}\right)}^{T}$, mixture models [16,17] are adopted to deal with the correlation and distribution of each subgroup of genes across species. An additional advantage of mixture models is that, after prior weights for the components are specified, estimates of the posterior probabilities of population membership can be formed for each observation to give a probabilistic clustering. As a result, the pooling of information for genes across species can be exploited to better understand the underlying relationship between the treatment intervention for both species.

Tailoring the mixture model to two-species experiments with restrictions on the parameters made according to Table 1 and assuming that the treatment effects for non-differentially expressed genes are deterministically zero, i.e., ${\left(\beta_{1a_{i}},\beta_{1h_{i}}\right)}^{T}={\left(0,0\right)}^{T}$, the following bivariate normal mixture model is adopted as the prior distribution of the vector ${\left(\beta_{1a_{i}},\beta_{1h_{i}}\right)}^{T}$:

(3) $$\begin{array}{ll} \;\left(\begin{array}{c} \beta_{1a_{i}}\\ \beta_{1h_{i}} \end{array}\right) & ∼\pi_{0}N\left(\begin{array}{cc} \left(\begin{array}{c} \mu_{a0}\\ \mu_{h0} \end{array}\right), & \left(\begin{array}{cc} \eta_{a0}^{2} & \rho_{0}\eta_{a0}\eta_{h0}\\ \rho_{0}\eta_{a0}\eta_{h0} & \eta_{h0}^{2} \end{array}\right) \end{array}\right)\\ & \;+\pi_{1}N\left(\begin{array}{cc} \left(\begin{array}{c} \mu_{a1}\\ \mu_{h1} \end{array}\right), & \left(\begin{array}{cc} \eta_{a1}^{2} & \rho_{1}\eta_{a1}\eta_{h1}\\ \rho_{1}\eta_{a1}\eta_{h1} & \eta_{h1}^{2} \end{array}\right) \end{array}\right)\\ & \;+\pi_{2}N\left(\begin{array}{cc} \left(\begin{array}{c} \mu_{a2}\\ \mu_{h2} \end{array}\right), & \left(\begin{array}{cc} \eta_{a2}^{2} & \rho_{2}\eta_{a2}\eta_{h2}\\ \rho_{2}\eta_{a2}\eta_{h2} & \eta_{h2}^{2} \end{array}\right) \end{array}\right)\\ & \;+\pi_{3}N\left(\begin{array}{cc} \left(\begin{array}{c} \mu_{a3}\\ \mu_{h3} \end{array}\right), & \left(\begin{array}{cc} \eta_{a3}^{2} & \rho_{3}\eta_{a3}\eta_{h3}\\ \rho_{3}\eta_{a3}\eta_{h3} & \eta_{h3}^{2} \end{array}\right) \end{array}\right)\\ & \;+\pi_{4}N\left(\begin{array}{cc} \left(\begin{array}{c} \mu_{a4}\\ \mu_{h4} \end{array}\right), & \left(\begin{array}{cc} \eta_{a4}^{2} & \rho_{4}\eta_{a4}\eta_{h4}\\ \rho_{4}\eta_{a4}\eta_{h4} & \eta_{h4}^{2} \end{array}\right) \end{array}\right)\\ & \;+\pi_{5}N\left(\begin{array}{cc} \left(\begin{array}{c} \mu_{a5}\\ \mu_{h5} \end{array}\right), & \left(\begin{array}{cc} \eta_{a5}^{2} & \rho_{5}\eta_{a5}\eta_{h5}\\ \rho_{5}\eta_{a5}\eta_{h5} & \eta_{h5}^{2} \end{array}\right) \end{array}\right)\\ & \;+\pi_{6}N\left(\begin{array}{cc} \left(\begin{array}{c} \mu_{a6}\\ \mu_{h6} \end{array}\right), & \left(\begin{array}{cc} \eta_{a6}^{2} & \rho_{6}\eta_{a6}\eta_{h6}\\ \rho_{6}\eta_{a6}\eta_{h6} & \eta_{h6}^{2} \end{array}\right) \end{array}\right)\\ & \;+\pi_{7}N\left(\begin{array}{cc} \left(\begin{array}{c} \mu_{a7}\\ \mu_{h7} \end{array}\right), & \left(\begin{array}{cc} \eta_{a7}^{2} & \rho_{7}\eta_{a7}\eta_{h7}\\ \rho_{7}\eta_{a7}\eta_{h7} & \eta_{h7}^{2} \end{array}\right) \end{array}\right)\\ & \;+\pi_{8}N\left(\begin{array}{cc} \left(\begin{array}{c} \mu_{a8}\\ \mu_{h8} \end{array}\right), & \left(\begin{array}{cc} \eta_{a8}^{2} & \rho_{8}\eta_{a8}\eta_{h8}\\ \rho_{8}\eta_{a8}\eta_{h8} & \eta_{h8}^{2} \end{array}\right) \end{array}\right), \end{array}$$

where *π*_*k*_ is the probability that an observation belongs to the *k*th component, with $\sum_{k=0}^{8}\pi_{k}=1\text{and}\pi_{k}\geq 0.$ The following restriction of the parameter space is imposed: *μ*_*a*0_ = 0, *μ*_*h*0_ = 0, *μ*_*a*1_ ≥ 0, *μ*_*h*1_ ≥ 0, *μ*_*a*2_ ≤ 0, *μ*_*h*2_ ≤ 0, *μ*_*a*3_ ≥ 0, *μ*_*h*3_ ≤ 0, *μ*_*a*4_ ≤ 0, *μ*_*h*4_ ≥ 0, *μ*_*a*5_ = 0, *μ*_*h*5_ ≥ 0, *μ*_*a*6_ = 0, *μ*_*h*6_ ≤ 0, *μ*_*a*7_ ≥ 0, *μ*_*h*7_ = 0, *μ*_*a*8_ ≤ 0, *μ*_*h*8_ = 0, *η*_*a*0_ = 0, *η*_*h*0_ = 0, *η*_*a*5_ = 0, *η*_*a*6_ = 0, *η*_*h*7_ = 0, *η*_*h*8_ = 0, *ρ*_0_ = 0, *ρ*_5_ = 0, *ρ*_6_ = 0, *ρ*_7_ = 0, and *ρ*_8_ = 0.

According to the theory of least squares, the marginal distribution of ${\left({\hat{\beta }}_{1a_{i}},{\hat{\beta }}_{1h_{i}}\right)}^{T}$, the parameter estimates, has means equal to the prior means of ${\left(\beta_{1a_{i}},\beta_{1h_{i}}\right)}^{T}$ and variances involving contributions from the prior distribution of ${\left(\beta_{1a_{i}},\beta_{1h_{i}}\right)}^{T}$ and the conditional distribution of ${\left({\hat{\beta }}_{1a_{i}},{\hat{\beta }}_{1h_{i}}\right)}^{T}$ given ${\left(\beta_{1a_{i}},\beta_{1h_{i}}\right)}^{T}$. The marginal distribution of ${\left({\hat{\beta }}_{1a_{i}},{\hat{\beta }}_{1h_{i}}\right)}^{T}$ is as follows:

(4) $$\begin{array}{ll} \;\left(\begin{array}{c} {\hat{\beta }}_{1a_{i}}\\ {\hat{\beta }}_{1h_{i}} \end{array}\right) & ∼\pi_{0}N\left(\begin{array}{cc} \left(\begin{array}{c} 0\\ 0 \end{array}\right), & \left(\begin{array}{cc} \sigma_{a0}^{2} & 0\\ 0 & \sigma_{h0}^{2} \end{array}\right) \end{array}\right)\\ & \;+\pi_{1}N\left(\begin{array}{cc} \left(\begin{array}{c} \mu_{a1}\\ \mu_{h1} \end{array}\right), & \left(\begin{array}{cc} \sigma_{a1}^{2} & \rho_{1}\sigma_{a1}\sigma_{h1}\\ \rho_{1}\sigma_{a1}\sigma_{h1} & \sigma_{h1}^{2} \end{array}\right) \end{array}\right)\\ & \;+\pi_{2}N\left(\begin{array}{cc} \left(\begin{array}{c} \mu_{a2}\\ \mu_{h2} \end{array}\right), & \left(\begin{array}{cc} \sigma_{a2}^{2} & \rho_{2}\sigma_{a2}\sigma_{h2}\\ \rho_{2}\sigma_{a2}\sigma_{h2} & \sigma_{h2}^{2} \end{array}\right) \end{array}\right)\\ & \;+\pi_{3}N\left(\begin{array}{cc} \left(\begin{array}{c} \mu_{a3}\\ \mu_{h3} \end{array}\right), & \left(\begin{array}{cc} \sigma_{a3}^{2} & \rho_{3}\sigma_{a3}\sigma_{h3}\\ \rho_{3}\sigma_{a3}\sigma_{h3} & \sigma_{h3}^{2} \end{array}\right) \end{array}\right)\\ & \;+\pi_{4}N\left(\begin{array}{cc} \left(\begin{array}{c} \mu_{a4}\\ \mu_{h4} \end{array}\right), & \left(\begin{array}{cc} \sigma_{a4}^{2} & \rho_{4}\sigma_{a4}\sigma_{h4}\\ \rho_{4}\sigma_{a4}\sigma_{h4} & \sigma_{h4}^{2} \end{array}\right) \end{array}\right)\\ & \;+\pi_{5}N\left(\begin{array}{cc} \left(\begin{array}{c} 0\\ \mu_{h5} \end{array}\right), & \left(\begin{array}{cc} \sigma_{a5}^{2} & 0\\ 0 & \sigma_{h5}^{2} \end{array}\right) \end{array}\right)\\ & \;+\pi_{6}N\left(\begin{array}{cc} \left(\begin{array}{c} 0\\ \mu_{h6} \end{array}\right), & \left(\begin{array}{cc} \sigma_{a6}^{2} & 0\\ 0 & \sigma_{h6}^{2} \end{array}\right) \end{array}\right)\\ & \;+\pi_{7}N\left(\begin{array}{cc} \left(\begin{array}{c} \mu_{a7}\\ 0 \end{array}\right), & \left(\begin{array}{cc} \sigma_{a7}^{2} & 0\\ 0 & \sigma_{h7}^{2} \end{array}\right) \end{array}\right)\\ & \;+\pi_{8}N\left(\begin{array}{cc} \left(\begin{array}{c} \mu_{a8}\\ 0 \end{array}\right), & \left(\begin{array}{cc} \sigma_{a8}^{2} & 0\\ 0 & \sigma_{h8}^{2} \end{array}\right) \end{array}\right), \end{array}$$

An EM algorithm is developed to accomplish the nontrivial likelihood maximization, along with methodology for handling singular covariance matrices that arise during the implementation of the algorithm. (See the Appendix for details). Gene membership is determined according to the maximum posterior probability that an observation ${\left({\hat{\beta }}_{1a_{i}},{\hat{\beta }}_{1h_{i}}\right)}^{T}$ comes from the *k*th component of the mixture.

## Simulation

The following Monte Carlo simulation studies investigated the performance of the proposed mixture model using information across two species in comparison to the traditional microarray method using just one-species information when identifying genes associated with treatment stimulus under several different scenarios.

Several factors influence the sampling properties of the estimated treatment effects ${\left({\hat{\beta }}_{1a_{i}},{\hat{\beta }}_{1h_{i}}\right)}^{T}$ using the mixture model were considered in the simulation:

• Replicates (number of arrays) per treatment for each species: *n*_*a*_ and *n*_*h*_ for animals and humans, respectively.

• Number of orthologous genes in each category: *n*_*k*_, *k* = 0,…,8, the *k*th category.

• Array noise: $e_{a_{\text{ij}}}$ and $e_{h_{\text{ij}}}$ in (1) and (2). Also recall that, by assumption, $e_{a_{\text{ij}}}$ and $e_{h_{\text{il}}}$ are independent $N(0,\sigma_{a}^{2})$ and $N(0,\sigma_{h}^{2})$ random variables.

• Parameters in (3) by which the sampling distribution of ${\left({\hat{\beta }}_{1a_{i}},{\hat{\beta }}_{1h_{i}}\right)}^{T}$ is determined.

With so many variables, it is impractical to study the sampling properties of the fitted model without fixing some variables. That is, an experimental design for the simulation in which these factors are completely considered is not feasible. The simulation study instead focused on three aspects. First, although high-density microarrays provide useful genome-wide data, they are often associated with a substantial amount of experimental noise that could affect the performance of the analysis. Hence, it is of interest to investigate how the array noise would affect the model efficiency on gene identification across species.

Second, the sample size of cross-species experiment is likely to be different, and may be one of the deciding factors of the power associated with the modeling approach. In particular, the efficiency of gene identification, whether the proposed model gains power over one-species experiments through pooling information across species, should be examined carefully, especially when the sample size of the experiments is small.

Third, over-fitting may be of concern. The proposed mixture model is, by its nature, highly structured and data driven. If the data are not driven by all nine categories as the model suggests, will the mixture model fail? Is the proposed model flexible enough to handle different types of data structure? To examine the model performance systematically and to test if the proposed model will fail when too many components are used to fit the data where there are actually fewer clusters, two types of data were generated: all nine categories non-empty (case I) and some of the nine components empty (case II).

In simulation studies case I and case II, two methods of gene identification were implemented: the proposed mixture model, utilizing information across two different species, and the traditional *t* statistics adjusting for multiple comparisons based on single-species data. Five hundred data sets were generated for each different scenario under each simulation study.

### Parameter determination and data generation

Theoretically, the number of genes in category 0 (non-differentially expressed in both species) should dominate others, and every other category may comprise some genes. Orthology information from HomoloGene of the National Center for Biotechnology Information (NCBI) ( http://www.ncbi.nlm.nih.gov/homologene) and Mouse Genome Informatics (MGI) of Jackson Laboratory [18] and the practical experience gained from analysis of two-species gene expression experiments at GlasoSmithKline (GSK) was used as reference to determine a reasonable number of genes in each category.

The vectors of the number of genes in each category (*n*_0_,*n*_1_,…,*n*_8_)^*T*^ determined for simulation studies case I and case II are categories (6,000,30, 30,30,30,100,100,100,100)^*T*^ and categories (6,000,30, 30,0,0,100,0,100,0)^*T*^, respectively. For experiments across species, sample sizes may differ. Considering that this proposed bivariate method could benefit from pooling information across species, especially when the sample size is small, and the practical situation, two scenarios were implemented: the number of replicates per treatment for each species is equal and small, and the number of replicates per treatment for animals is greater than humans. In addition, to evaluate how robust the proposed method is against array noise, two situations were considered: the two experiments are equally noisy and the human data are noisier than the animal’s. Furthermore, values of parameters in (3) for the sampling distribution of ${\left({\hat{\beta }}_{1a_{i}},{\hat{\beta }}_{1h_{i}}\right)}^{T}$ were predetermined. Variances of ${\left(\beta_{1a_{i}},\beta_{1h_{i}}\right)}^{T}$ in each component were assumed to be the same: $\eta_{a1}^{2}=\eta_{a2}^{2}=\eta_{a3}^{2}=\eta_{a4}^{2}=\eta_{a7}^{2}=\eta_{a8}^{2}=\eta_{h1}^{2}=\eta_{h2}^{2}=\eta_{h3}^{2}=\eta_{h4}^{2}=\eta_{h5}^{2}=\eta_{h6}^{2}=0.25$. The correlation between ${\left(\beta_{1a_{i}},\beta_{1h_{i}}\right)}^{T}$ in categories (1,2,3,4) was assumed to be 0.9, i.e., *ρ*_1_ = *ρ*_2_ = *ρ*_3_ = *ρ*_4_ = 0.9. Nonzero component means (*μ*_*ak*_,*μ*_*hk*_)^*T*^, *k* = 0,…,8, were determined so that |*μ*/*η*| = 0.5 or 1.5. The combination of these parameters resulted in eight different scenarios for each case as presented in Table 2. Note that $\left|\mu_{\beta_{1a_{i}}}\right|$ and $\left|\mu_{\beta_{1h_{i}}}\right|$ represent the absolute value of the mean vector of ${\left(\beta_{1a_{i}},\beta_{1h_{i}}\right)}^{T}$.

**Table 2:** Combination of parameters for simulation studies case I and case II

**Case I**	**sim1**	**sim2**	**sim3**	**sim4**	**sim5**	**sim6**	**sim7**	**sim8**
*n*_*a*_	10	10	10	10	100	100	100	100
*n*_*h*_	10	10	10	10	10	10	10	10
$\left|\mu_{\beta_{1a_{i}}}\right|$	0.25	0.75	0.25	0.75	0.25	0.75	0.25	0.75
$\left|\mu_{\beta_{1h_{i}}}\right|$	0.25	0.75	0.25	0.75	0.25	0.75	0.25	0.75
$\sigma_{a}^{2}$	0.1	0.1	0.1	0.1	0.1	0.1	0.1	0.1
$\sigma_{h}^{2}$	0.1	0.1	0.3	0.3	0.1	0.1	0.3	0.3
Case II	sim9	sim10	sim11	sim12	sim13	sim14	sim15	sim16

After generating ${\left(\beta_{1a_{i}},\beta_{1h_{i}}\right)}^{T}$ accordingly, the next step is to simulate the two species gene expression data *X*_*a*_ and *X*_*h*_ based on linear models (1) and (2). Note that $\beta_{0a_{i}}$ and $\beta_{0h_{i}}$ are independent *N*(8,1) random variables for differentially expressed genes and deterministically 0 for non-differentially expressed genes.

### Simulation results

It is of interest to compare how effective the mixture model is on gene identification using information across two species with the conventional one-species approach. The conventional two-sample *t* test for gene selection was performed using just single species data (animals or humans) and a multiplicity adjustment was made according to the procedure proposed by Benjamini and Hochberg [19].

The results are presented in Table 3. The first section of Table 3, categories (1, 2, 3, 4, 5, 6), manifests the number of genes classified into categories (1, 2, 3, 4, 5, 6) using the the mixture model (Mixture) and the number of genes selected using human data only (Human only), with the corresponding nominal FDR controlled at FDR_*I*_. For each simulated data set, FDR_*I*_ was calculated as (number of genes that are erroneously classified into categories (1, 2, 3, 4, 5, 6))/(total number of genes classified into categories (1, 2, 3, 4, 5, 6)). This was to ensure a fair comparison between the mixture model and the conventional one-species method. FDR_*II*_ was calculated in the same fashion. Avg FDR_*I*_ and Avg FDR_*II*_ are simply the averaged values of FDR_*I*_ and FDR_*II*_ across the 500 simulated data sets. When the estimated nominal FDR = 0, i.e., the mixture model did not falsely categorize any genes, the nominal FDR for the single-species method was controlled at 0.0001. The second section of Table 3, categories (1,2,3,4,7,8), represents the number of genes classified into categories (1,2,3,4,7,8) using the mixture model (Mixture) and number of genes selected using animal data alone (Animal only). Beneath each set of eight simulation cases is Tukey’s Honestly Significant Difference (HSD) for a familywise error rate of 0.05 for the results obtained using the proposed mixture model. Tukey’s HSD was calculated as *q*_0.05_(8,3,992) × (MS(Error) /500)^1/2^, since there were eight simulation cases (500 simulated data sets in each situation) in each simulation study (case I and case II) and the error degree of freedom was 3,992. MS(Error) denotes the error mean square (= Error sum of squares/Error degree of freedom, see Table 4). *q*_0.05_(8,3,992) = 4.29.

**Table 3:** The number of genes selected based on (a) bivariate mixture model, (b) conventional one-species approach

	**Categories (1,2,3,4,5,6)**			**Categories (1,2,3,4,7,8)**		
	**Mixture**	**Avg FDR**_***I***_	**Human only**	**Mixture**	**Avg FDR**_***II***_	**Animal only**
sim1	132	0.034	100(0.070)	129	0.029	96(0.063)
sim2	224	0.003	145(0.021)	223	0.012	188(0.016)
sim3	113	0.246	85(0.318)	135	0.048	109(0.073)
sim4	166	0.050	115(0.043)	222	0.012	188(0.016)
sim5	132	0.028	98(0.041)	234	0.004	238(0.012)
sim6	227	0.011	194(0.021)	289	0.003	289(0.007)
sim7	112	0.235	78(0.282)	241	0.011	246(0.020)
sim8	167	0.048	124(0.065)	288	0.002	288(0.007)
Tukey’s HSD	30.908	0.021		52.958	0.016	
sim9	92	0.126	81(0.296)	88	0.129	75(0.307)
sim10	118	0.033	104(0.048)	118	0.036	99(0.061)
sim11	141	0.470	109(0.670)	80	0.128	70(0.214)
sim12	103	0.116	68(0.118)	118	0.049	102(0.088)
sim13	84	0.140	67(0.169)	180	0.349	167(0.407)
sim14	120	0.023	88(0.023)	152	0.022	151(0.101)
sim15	119	0.480	99(0.636)	168	0.358	157(0.369)
sim16	96	0.093	57(0.105)	147	0.020	148(0.061)
Tukey’s HSD	3.947	0.011		2.337	0.004	

Under simulation studies case I and case II. Numbers in parentheses are the observed FDRs. Averaged over the 500 simulated datasets. Tukey’s HSD for an *α* level of 0.05 is included beneath each set of eight simulation cases.

**Table 4:** ANOVA table to quantify variability

			**Categories (1, 2, 3, 4, 5, 6)**	**Categories (1, 2, 3, 4, 7, 8)**
	**Source of variation**	***df***	**Sum of squares**	**Sum of squares**
Case I	Replicates	1	1,141(0.006)	7,319,401(0.408) ^∗^
	Mean magnitude	1	5,415,341(11.560) ^∗^	4,982,736(0.252) ^∗^
	Array noise	1	1,561,198(15.950) ^∗^	6,353(0.042)
	Replicates × Mean magnitude	1	1,197(0.032)	392,099(0.122) ^∗^
	Replicates × Array noise	1	697(0.012)	351(0.011)
	Mean magnitude × Array noise	1	400,720(7.021) ^∗^	16,601(0.043) ^∗^
	Replicates × Mean magnitude × Array noise	1	145(0.002)	214(0.010)
	Error	3,992	1,689,667(12.887)	592,184(1.817)
	Total	3,999	9,070,107(47.471)	13,309,941(2.706)
Case II	Replicates	1	73,917(0.006)	3,676,664(2.888)
	Mean magnitude	1	6(56.346)	22,274(2.830)
	Array noise	1	130,794(43.770) ^∗^	865,448(0.001) ^∗^
	Replicates × Mean magnitude	1	37,277(0.190)	36,778(1.038)
	Replicates × Array noise	1	34,404(0.015)	5,065(0.049)
	Mean magnitude × Array noise	1	929,915(17.537)	19,128(0.043)
	Replicates × Mean magnitude × Array noise	1	1,467(0.004)	58(0.000)
	Error	3,992	103,604,971(47.182)	304,161,196(27.427)
	Total	3,999	104,812,751(165.052)	308,786,610(34.276)

ANOVA was performed independently for simulation studies case I and case II to quantify the variability among the results (gene counts and observed FDRs). Numbers in parentheses are the sum of squares for the observed FDRs. For gene counts, *indicates which sources of variability could be declared significant at level *α* = 0.05.

The effect of array noise on the mixture model can be easily seen from the column of Avg FDR_*I*_ and by comparing the results of sim1 vs. sim3, sim5 vs. sim7, sim9 vs. sim11, and sim13 vs. sim15. These are the cases with smaller means and variances for humans which change from 0.1 to 0.3 for each pair of comparisons. The differences between the observed FDRs for these four groups were at least 0.2 and 0.3 for case I and case II, respectively. In contrast, when means were larger, changes of variances did not seem to affect the results in the sense that the corresponding observed FDRs had barely changed while the variances of human increased. The observed FDRs for animals were not as sensitive to the array noise on humans as the observed FDRs for humans.

Under both simulation studies case I and case II, increasing the number of replicates in the animal experiment helped the gene identification for animals: more animal genes were identified for sim5 to sim8 than for sim1 to sim4, and for sim13 to sim16 than for sim9 to sim12. The corresponding FDRs were also lower for sim5 to sim8 and sim13 to sim16. In contrast, increasing the number of replicates in the animal experiment did not significantly improve the results of gene identification for humans.

Table 4 is the summary of a three-way analysis of variance (ANOVA). Three factors, each with two levels, were used in the analysis: replicates ((*n*_*a*_,*n*_*h*_)^*T*^ = (10,10)^*T*^ or (100,10)^*T*^), mean magnitude ( ${\left(|\mu_{\beta_{1a_{i}}}|,|\mu_{\beta_{1h_{i}}}|\right)}^{T}={\left(0.25,0.25\right)}^{T}$ or (0.75,0.75)^*T*^), and array noise ( ${\left(\sigma_{a}^{2},\sigma_{h}^{2}\right)}^{T}={\left(0.1,0.1\right)}^{T}$ or (0.1,0.3)^*T*^). This analysis, performed independently, quantifies the variability among the results (gene counts and observed FDRs) obtained using the proposed mixture model in Table 3 for the 16 different simulated situations under simulation studies case I and case II.

Throughout the 16 simulation cases, with nominal FDR controlled at FDR_*I*_, the bivariate mixture model outperformed the single-species method for human gene identification by always recognizing more genes with lower observed FDRs. For the animal part, the mixture model performed at least as well as the single-species method on gene selection by identifying at least as many genes. Notice that selecting genes related to humans (categories (1, 2, 3, 4, 5, 6)) seemed to be associated with higher false discovery rate than selecting genes related to animals (categories (1, 2, 3, 4, 7, 8)). Furthermore, the observed FDRs were lower for case I than for case II, regardless of the type of genes interested (differentially expressed for humans or animals).

The comprehensive simulation study suggested that the proposed model, though highly structured, offered advantages over single-species analyses, especially when the magnitude of differential expression due to different treatment intervention was weak.

## Application: the mouse and human type II diabetes experiment

### Background introduction

A systems biology study was completed by GlaxoSmithKline (GSK) to study the efficacy of type II diabetes drugs in both preclinical (mice) and clinical (humans) experiments. The mouse and human data were collected and preprocessed using Affymetrix MAS 5.0 at the probe set level on Affymetrix MOE430A array and Human Genome U133 Plus 2.0 array, respectively. For mice, the total number of probe sets was 22,690. Mice were fed a diet enriched in fat (58% kcal from fat) for 8 weeks prior to treatment. Most of the mice of this susceptible strain developed obesity and mild hyperglycemia and hyperinsulinemia. Control mice on an 11% low fat diet remained normal. The mouse treatment arm consisted of a diabetes drug at multiple dose levels with vehicle controls over a 2-week period. The study was a full factorial design, where 40 animals in high fat diet and 40 animals in low fat diet were randomized to receive either placebo, or different dosages of the type II diabetes drug (low, medium or high). Note that the results for mice were measured at one time point, the end of the study. For simplicity, only integration results on animals treated with placebo (10 mice) or high dose of the drug (9 mice) were demonstrated.

On the other hand, there were 54,676 probe sets in the human data set and all 59 subjects were type II diabetes patients. The gene expression measurements for human subjects were collected twice during the experiment, one at baseline before the treatment started (week 0) and the other one at the completion of the study (week 8). Among the 59 subjects, 14 only had data for only one time point and hence were not included in further data analysis. Subjects in the clinical trial were treated by either placebo, or three other type II diabetes drugs, including the one given to the mice. Titrated dosing was implemented to ensure that each person received his/her dose based on his/her body profile. Human subjects treated with placebo (number of subjects = 11) and the same type II diabetes drug given to mice (number of subjects = 13) were used in this analysis. Besides being measured at two time points, other information contained in the human data and used in the data analysis included: prior therapy (four categorical levels) and concomitant medication status (two categorical levels).

Data for both species were logarithmic transformed (base equal to 2). Methods for combining information from multiple probe sets that were not identical have been discussed in many publications [20,21]. A gene-level transcript value in order to pair the mouse and human genes through orthology was obtained by averaging probe sets across a gene. This resulted in 13,483 and 20,252 genes for mouse and human, respectively. Missing values were replaced with array means in both data sets.

The mouse and human orthology information, MGI release 4.32, was used to map the mouse and human orthologous genes. Human and mouse orthologs (17,834) were included in release 4.32. Combining the information among MGI release 4.32 and mouse and human genes from the GSK data gave a total of 11,922 orthologs.

In addition to the measurements of gene expression for both mice and humans, different efficacy endpoints, such as blood glucose, insulin, hemoglobin A1c (HbA1c), and others were also measured during the preclinical and clinical experiments by GSK in order to evaluate the effect of treatment intervention on both species.

The purpose of this data analysis was to evaluate the capability of the proposed mixture model, an approach utilizing the information across two species, to identify genes that may help scientists reveal the biological similarity between two species (ex: mouse and human), essentially genes in categories (1,2,3,4), and so to improve the efficiency of drug development by decreasing the compound attrition rate from preclinical trials to clinical trials.

### Data analysis

The estimated treatment effects for mice and humans are obtained by the following independent simple linear models:

(5) $$\begin{array}{l} X_{a_{\text{ij}}}=\beta_{0a_{i}}+\beta_{1a_{i}}T_{a_{j}}+e_{a_{\text{ij}}}, \end{array}$$

(6) $$\begin{array}{ll} \;X_{h_{\text{il}}}^{\text{week}8} & =\beta_{0h_{i}}+X_{h_{\text{il}}}^{\text{week}0}+\beta_{1h_{i}}T_{h_{l}}+\beta_{2h_{i}}\;\text{PriorTherapy}\\ & \;+\beta_{3h_{i}}\text{ConMed}+e_{h_{\text{il}}}, \end{array}$$

where *i* = 1,…,11,922 (number of orthologs); *j* = 1,…,19 (number of mice); *l* = 1,…,24 (number of humans). $X_{a_{\text{ij}}}$, $X_{h_{\text{il}}}^{\text{week}0}$ and $X_{h_{\text{il}}}^{\text{week}8}$ are gene expressions from the *i*th orthologous gene for the *j*th mouse, gene expression from the *i*th orthologous gene for the *l*th human before treatment intervention, and gene expression from the *i*th orthologous gene for the *l*th human at the completion of the clinical experiment, respectively. $T_{a_{j}}$ and $T_{h_{l}}$ are {0,1} treatment indicators, and $e_{a_{\text{ij}}}$ and $e_{h_{\text{il}}}$ are independent $N(0,\sigma_{a}^{2})$ and $N(0,\sigma_{h}^{2})$ random variables. Additionally, there are two more covariates in the human model: PriorTherapy (a four-level categorical variable indicating patients’ therapy prior to the clinical trial) and ConMed (a two-level categorical variable for concomitant medication status).

Equation (4) was used to model the distribution of the estimated treatment effects. Genes in categories (1, 2, 3, 4) are believed to be potential biomarkers that can greatly improve the design of the process of drug development as these orthologous genes interact with drugs in a way that shows some relationship between two different species (in this case, human and mouse) and so studying the behavior of these genes in preclinical trials might help scientists better understand the mechanism of drug activities in clinical trials.

### Results

#### Parameter estimation

The maximum likelihood estimates of the parameters in the bivariate normal mixture model using the EM algorithm are given in Table 5. The estimated mixture weight for category 0 was ${\hat{\pi }}_{0}=0.889$ indicating approximately 10,599 (11,922 × 0.889) pairs of uninteresting mouse and human orthologs. (*μ*_*a*_,*μ*_*h*_)^*T*^ denotes the mean vector of each mixture component. The estimated treatment effect means of mice were in general larger than that of humans, indicating that the magnitude of the difference of expression in genes due to treatment intervention in mice tended to be larger than in genes where differential expression is exhibited in humans. The estimated variance for mice ${\hat{\sigma }}_{\text{ak}}^{2}$ tended to be larger than that for humans ${\hat{\sigma }}_{\text{hk}}^{2}$, suggesting that overall, the variability in the mouse data set was larger than the human data set. The estimated correlation coefficients between the treatment effects for both species were (-0.531,0.380,-1,0.102) for categories (1,2,3,4), respectively. The estimated correlation for category 3 was based on only two observations. Standard errors of the parameter estimates from the two-species experiment were obtained by bootstrapping with 1,000 bootstrap replicates. Based on the bootstrap standard errors, there was little evidence of bias for the parameter estimates.

**Table 5:** Parameter estimates of the bivariate mixture model

	**Parameter estimates**					
**Category**	${\hat{\pi }}_{k}$	${\hat{\mu }}_{\text{ak}}$	${\hat{\mu }}_{\text{hk}}$	${\hat{\sigma }}_{\text{ak}}^{2}$	$\hat{\rho_{k}\sigma_{\text{ak}}\sigma_{\text{hk}}}$	${\hat{\sigma }}_{\text{hk}}^{2}$
0	0.889(0.013)	NE	NE	0.117(0.002)	NE	0.052(0.001)
1	0.002(0.001)	1.440(0.190)	0.139(0.203)	0.112(0.064)	-0.070(0.040)	0.154(0.061)
2	0.001(0.001)	-1.174(0.228)	-0.853(0.128)	0.156(0.132)	0.033(0.053)	0.048(0.039)
3	0.000(0.001)	2.104(0.319)	-1.231(0.361)	0.006(0.124)	-0.005(0.078)	0.003(0.111)
4	0.002(0.001)	-1.047(0.081)	0.660(0.128)	0.027(0.018)	-0.005(0.023)	0.087(0.047)
5	0.021(0.001)	NE	0.669(0.003)	0.144(0.000)	NE	0.050(0.006)
6	0.049(0.002)	NE	-0.787(0.001)	0.149(0.000)	NE	0.055(0.004)
7	0.003(0.008)	1.528(0.310)	NE	0.543(0.201)	NE	0.084(0.022)
8	0.034(0.009)	-1.038(0.146)	NE	0.225(0.051)	NE	0.055(0.006)

Bootstrap (*B* = 1,000) standard errors in parentheses. NE, not estimated.

#### Gene identification

**Figure 1:**
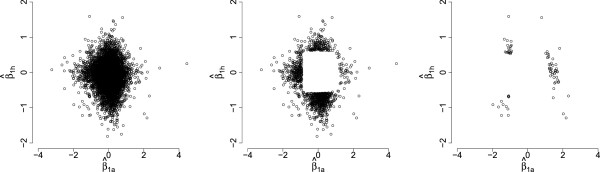
**Scatter plots of the estimated treatment effects**${\left({\hat{\beta }}_{1a_{i}},{\hat{\beta }}_{1h_{i}}\right)}^{T}$** before and after gene membership identification.** From left to right: all orthologs, orthologs differentially expressed in either species (categories (1,2,3,4,5,6,7,8)), and orthologs differentially expressed in both species (categories (1,2,3,4)).

The vector of the number of genes identified for each category was ${\left({\hat{n}}_{0},{\hat{n}}_{1},\ldots ,{\hat{n}}_{8}\right)}^{T}$ = (10,814, 41, 12, 2, 20, 168, 578, 12, 275)^*T*^. Figure 1 displays the scatter plots of ${\left({\hat{\beta }}_{1a_{i}},{\hat{\beta }}_{1h_{i}}\right)}^{T}$ before and after gene membership identification, including the scatter plot of ${\left({\hat{\beta }}_{1a_{i}},{\hat{\beta }}_{1h_{i}}\right)}^{T}$ for all orthologs, orthologs after eliminating the uninteresting ones (orthologs not in category 0), and orthologs reacting to the treatment stimulus for both species (orthologs in categories (1, 2, 3, 4)).

Based on the bivariate mixture model, among the 11,922 pairs of orthologs, 10,814 pairs did not react to the drug treatment for either species, 75 pairs of orthologs (sum of the gene counts in categories 1 through 4) showed evidence of differentiation between treatments for both species. Genes in categories (5,6,7,8) are also potential candidates for further investigation to improve the process of drug development since studying these genes might uncover the myth of the overall high attrition rate of a drug compound from preclinical trials to clinical trials.

**Figure 2:**
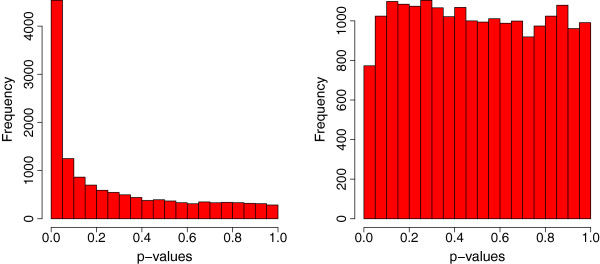
**Histograms of *p * values from tests of no treatment effects.** From left to right: *p* value histogram for mice and *p* value histogram for humans.

In comparison, an attempt was made to identify differentially expressed human genes using solely the human data, i.e., traditional *t* statistics were used to test whether or not $\beta_{1h_{i}}=0$ and the approach of Benjamini and Hochberg [19] was used to adjust for multiple comparisons. With the nominal FDR controlled at 0.01, 0.1, 0.5, and 0.9, this single-species method failed to identify any differentially expressed human genes at any levels of FDR. With the *p* values histograms in Figure 2 showing an obvious difference between the observed significance of differential expression for mice and humans, specifically, the *p* values were nearly uniformly distributed for humans, the result was not surprising.

## Concluding remarks

This research was motivated by a fundamental yet still not well-understood problem in the drug development process. The results obtained in preclinical animal trials do not seem to translate well enough to make inferences for human clinical trials, resulting in an undesirably high attrition rate in human experiments. A bivariate mixture model which utilizes information across two species was proposed to identify genes that exhibit similar patterns of expression across species, with the hope that studying genes could help understand biological differences across species at the molecular level and ultimately help reduce attrition in drug development. It is also of great interest to identify genes active in animal, but not in human since studying this group of genes might lead to answers that explain why some drug trials fail in translation to humans. The comprehensive simulation study suggested that the proposed model, though highly structured, can accommodate various configurations of differential gene expression, especially when the magnitude of differential expression due to different treatment intervention was weak.

In the application of the bivariate mixture model on the GSK type II diabetes experiment, the mixture model was able to separate differentially expressed genes from non-differentially expressed genes. A potential multi-gene predictor may be developed according to the genes identified by the bivariate mixture model to benefit patients in therapeutic decision making.

The mixture model is highly structured, with strong but somewhat simplifying assumptions. The grouping of all genes for which the expression difference is positive in both species into a single category parameterized by a single bivariate mean may be somewhat of a simplistic approach. In practice, the data may not be normally distributed or be comprised by exactly nine groups and lead to bias and inefficiency. Forcing the normality assumption and the grouping may be inefficient. However, by modeling the least squares estimates of the expression differences as bivariate realizations from a distribution with a single mean vector, some flexibility in the model is retained. It is perhaps less important to precisely quantify the magnitude of the expression difference than to determine whether it is positive or negative and whether or not the direction is preserved across species.

Using the proposed mixture model for gene identification is completely data-driven. The simulation study in Section ‘Simulation’ indicated that the mixture model at times report a poor observed false discovery rate due to large variability in measurement of expression. Currently, gene membership classification is determined by maximizing the posterior probability that observation *y*_*i*_ belongs to the *k*th cluster. It is possible to choose costs to attach weight to different types of misclassification and then choose a classification rule to minimize expected cost. This rule may lead to a more desirable list that can better accommodate goals for future research.

The mixture model currently only handles data with two treatments/cancer types/drugs, i.e., data containing only two-level variables. In practice, it is often the case that an experiment is run under multiple conditions. An extension of the model to handle experiments with factors with three-or-more levels is an important future work.

This approach focuses on identifying orthologs that show same/opposite mechanism between two species. However, genes identified by the bivariate mixture model (genes in categories (1, 2, 3, 4)) may not lead to the most powerful model for prediction of cancer types/response status for either one of the species. The most powerful model may be based on those human genes that lie in categories 5 and 6. For those genes, the corresponding animal genes show no signs of differential expression. Hence, incorporating prediction ability into the model or developing a prediction model that can help utilize the genes selected by the mixture model is of great interest and is an obvious candidate for future research.

## Appendix

### Mixture models and the EM algorithm

Estimating the parameters in (4) is nontrivial. Redner and Walker [22] offer an excellent review of estimating the parameters which determine a mixture density. In particular, the paper is devoted to a particular iterative procedure for numerically approximating maximum likelihood estimates (MLE) of the parameters in mixture densities. This method was formalized by Dempster et al. [23] and termed the *EM(Expectation-Maximization)algorithm*, and is used for numerically approximating the maximum likelihood estimates for (4).

#### The EM algorithm with no constraints

For a finite mixture model with *C* components, given data **y** with independent multivariate observations **y**_1_,…,**y**_*g*_, each **y**_*i*_ is taken to be a realization of the mixture probability density function,

$$f({\text{y}}_{i}|\Psi )=\overset{C}{\underset{k=1}{\sum }}\pi_{k}\;f_{k}({\text{y}}_{i}|\theta_{k}),$$

where **Ψ** = (***θ***_1_,…, ***θ***_*C*_,*π*_1_,…,*π*_*C*_)^*T*^, a vector of unknown parameters. *f*_*k*_ and ***θ***_*k*_ are the density and parameters of the *k*th component in the mixture, respectively. *π*_*k*_ is the probability that an observation arises from the *k*th component. Note that *π*_*k*_ ≥ 0 and $\sum_{k=1}^{C}\pi_{k}=1$. For classification purpose and to achieve minimum misclassification rates, **y**_*i*_ is assigned to the population (category) for which the posterior probability that **y**_*i*_ belongs to the *k*th cluster (the *k*th component of the mixture) is maximized. The posterior probability is given by

$$\tau_{k}({\text{y}}_{i};\Psi )=\frac{\pi_{k}\;f_{k}({\text{y}}_{i}|\theta_{k})}{\sum_{h=1}^{C}\pi_{h}f_{h}({\text{y}}_{i}|\theta_{h})}.$$

In summary, the EM algorithm may be implemented to maximize the likelihood of a multivariate normal mixture model by following these two steps:

***E-step*:** *The E-step on the (j+1) iteration takes the conditional expectation of the complete-data log likelihood, given the observed data (Q(***Ψ**|**Ψ**^(*j*)^)).

At the (*j* + 1) iteration, the E-step results in

(7) $$Q(\Psi |\Psi^{\left(j\right)})=\overset{g}{\underset{i=1}{\sum }}\overset{C}{\underset{k=1}{\sum }}\;\tau_{k}({\text{y}}_{i};\Psi^{\left(j\right)})(\log\pi_{k}+\log f_{k}({\text{y}}_{i}|\theta_{k})),$$

where $f_{k}({\text{y}}_{i}|\theta_{k})={\left(2\pi \right)}^{-\frac{p}{2}}{\left|\Sigma_{k}\right|}^{-\frac{1}{2}}e^{-\frac{1}{2}{\left({\text{y}}_{i}-\mu_{k}\right)}^{T}\Sigma_{k}^{-1}({\text{y}}_{i}-\mu_{k})}$ and $\tau_{k}({\text{y}}_{i};\Psi^{\left(j\right)})=\frac{\pi_{k}^{\left(j\right)}f_{k}({\text{y}}_{i}|\theta_{k}^{\left(j\right)})}{\sum_{h=1}^{C}\pi_{h}^{\left(j\right)}f_{h}({\text{y}}_{i}|\theta_{h}^{\left(j\right)})}$.

***M-step*:** *The M-step on the (j+1) iteration requires the global maximization of Q*(**Ψ**|**Ψ**^(*j*)^) *with respect to***Ψ** *over the parameter space to give the updated estimate***Ψ**^(*j*+1)^.

(8) $${\hat{\pi }}_{k}^{\left(j+1\right)}=\frac{\sum_{i=1}^{g}\tau_{k}({\text{y}}_{i};\Psi^{\left(j\right)})}{g};$$

(9) $${\hat{\mu }}_{k}^{\left(j+1\right)}=\frac{\sum_{i=1}^{g}\tau_{k}({\text{y}}_{i};\Psi^{\left(j\right)}){\text{y}}_{i}}{\sum_{i=1}^{g}\tau_{k}({\text{y}}_{i};\Psi^{\left(j\right)})};$$

(10) $${\hat{\Sigma }}_{k}^{\left(j+1\right)}=\frac{\sum_{i=1}^{g}\tau_{k}({\text{y}}_{i};\Psi^{\left(j\right)}){\left({\text{y}}_{i}-\mu_{k}\right)}^{T}({\text{y}}_{i}-\mu_{k})}{\sum_{i=1}^{g}\tau_{k}({\text{y}}_{i};\Psi^{\left(j\right)})}.$$

The iterations of the EM algorithm continue until some stopping criterion is met, such as the difference of the conditional expectation of the complete-data log likelihood at the (*j* + 1) step and the conditional expectation of the complete-data log likelihood at the *j* step is sufficiently small, i.e.,

$$Q(\Phi^{\left(j+1\right)}|\Phi^{\left(j\right)})-Q(\Phi^{\left(\;j\right)}|\Phi^{\left(j\right)})\leq \varepsilon .$$

Throughout this research, *ε* = 0.0001.

#### The EM Algorithm with constraints

With constraints on the parameter space, the EM algorithm derived in Section ‘The EM algorithm with no constraints’ cannot be used directly. To accommodate the restricted parameter space introduced for (4), first note that (4) is a bivariate normal mixture with nine components and the density for each component can be written as

$$\begin{array}{l} f_{k}\left({\text{y}}_{i}|\theta_{k}\right)\\ =\frac{e^{-\frac{1}{2}\frac{1}{\sigma_{a_{k}}^{2}\sigma_{h_{k}}^{2}\left(1-\rho_{k}^{2}\right)}\left(\sigma_{h_{k}}^{2}{\left(y_{a_{i}}-\mu_{a_{k}}\right)}^{2}-2\rho_{k}\sigma_{a_{k}}\sigma_{h_{k}}\left(y_{a_{i}}-\mu_{a_{k}}\right)\left(y_{h_{i}}-\mu_{h_{k}}\right)+\sigma_{a_{k}}^{2}{\left(y_{h_{i}}-\mu_{h_{k}}\right)}^{2}\right)}}{2\pi \sqrt{\sigma_{a_{k}}^{2}\sigma_{h_{k}}^{2}\left(1-\rho_{k}^{2}\right)}}, \end{array}$$

where ${\text{y}}_{i}={\left(y_{a_{i}},y_{h_{i}}\right)}^{T}={\left({\hat{\beta }}_{1a_{i}},{\hat{\beta }}_{1h_{i}}\right)}^{T}$ and $\theta_{k}={\left(\mu_{a_{k}},\mu_{h_{k}},\;\sigma_{a_{k}}^{2},\sigma_{h_{k}}^{2},\rho_{k}\right)}^{T}$.

The solution for the E-step remains the same. The membership probability *τ*_*k*_(**y**_*i*_;**Ψ**^(*j*)^) can be obtained by taking the conditional expectation of the complete-data log likelihood, given the observed data.

As for the M-step, the MLE for *π*_*k*_ remains the same, ${\hat{\pi }}_{k}^{\left(j+1\right)}=\frac{\sum_{i=1}^{g}\tau_{k}({\text{y}}_{i};\Psi^{\left(j\right)})}{g}$. However, the MLEs for ***μ***_*k*_ and ***Σ***_*k*_ need to be modified according to the constrained parameter space for each mixture component.

Essentially, the MLEs on the (*j* + 1) iteration for (*μ*_*ak*_,*μ*_*hk*_)^*T*^, *k* = 1,…,4, are

$$\begin{array}{l} {\hat{\mu }}_{a1}^{\left(j+1\right)}=\left\{\begin{array}{ll} \frac{\sum_{i=1}^{g}\tau_{1}({\text{y}}_{i};\Psi^{\left(j\right)})y_{a_{i}}}{\sum_{i=1}^{g}\tau_{1}({\text{y}}_{i};\Psi^{\left(j\right)})} & \text{if}\sum_{i=1}^{g}\tau_{1}(y_{i};\Psi^{\left(j\right)})y_{a_{i}}\geq 0;\\ 0 & \text{otherwise.} \end{array}\right)\\ {\hat{\mu }}_{h1}^{\left(j+1\right)}=\left\{\begin{array}{ll} \frac{\sum_{i=1}^{g}\tau_{1}({\text{y}}_{i};\Psi^{\left(j\right)})y_{h_{i}}}{\sum_{i=1}^{g}\tau_{1}({\text{y}}_{i};\Psi^{\left(j\right)})} & \text{if}\sum_{i=1}^{g}\tau_{1}({\text{y}}_{i};\Psi^{\left(j\right)})y_{h_{i}}\geq 0;\\ 0 & \text{otherwise.} \end{array}\right)\\ {\hat{\mu }}_{a2}^{\left(j+1\right)}=\left\{\begin{array}{ll} \frac{\sum_{i=1}^{g}\tau_{2}({\text{y}}_{i};\Psi^{\left(j\right)})y_{a_{i}}}{\sum_{i=1}^{g}\tau_{2}({\text{y}}_{i};\Psi^{\left(j\right)})} & \text{if}\sum_{i=1}^{g}\tau_{2}({\text{y}}_{i};\Psi^{\left(j\right)})y_{a_{i}}\leq 0;\\ 0 & \text{otherwise.} \end{array}\right)\\ {\hat{\mu }}_{h2}^{\left(j+1\right)}=\left\{\begin{array}{ll} \frac{\sum_{i=1}^{g}\tau_{2}({\text{y}}_{i};\Psi^{\left(j\right)})y_{h_{i}}}{\sum_{i=1}^{g}\tau_{2}({\text{y}}_{i};\Psi^{\left(j\right)})} & \text{if}\sum_{i=1}^{g}\tau_{2}({\text{y}}_{i};\Psi^{\left(j\right)})y_{h_{i}}\leq 0;\\ 0 & \text{otherwise.} \end{array}\right)\\ {\hat{\mu }}_{a3}^{\left(j+1\right)}=\left\{\begin{array}{ll} \frac{\sum_{i=1}^{g}\tau_{3}({\text{y}}_{i};\Psi^{\left(j\right)})y_{a_{i}}}{\sum_{i=1}^{g}\tau_{3}({\text{y}}_{i};\Psi^{\left(j\right)})} & \text{if}\sum_{i=1}^{g}\tau_{3}({\text{y}}_{i};\Psi^{\left(j\right)})y_{a_{i}}\geq 0;\\ 0 & \text{otherwise.} \end{array}\right)\\ {\hat{\mu }}_{h3}^{\left(j+1\right)}=\left\{\begin{array}{ll} \frac{\sum_{i=1}^{g}\tau_{3}({\text{y}}_{i};\Psi^{\left(j\right)})y_{h_{i}}}{\sum_{i=1}^{g}\tau_{3}({\text{y}}_{i};\Psi^{\left(j\right)})} & \text{if}\sum_{i=1}^{g}\tau_{3}({\text{y}}_{i};\Psi^{\left(j\right)})y_{h_{i}}\leq 0;\\ 0 & \text{otherwise.} \end{array}\right)\\ {\hat{\mu }}_{a4}^{\left(j+1\right)}=\left\{\begin{array}{ll} \frac{\sum_{i=1}^{g}\tau_{4}({\text{y}}_{i};\Psi^{\left(j\right)})y_{a_{i}}}{\sum_{i=1}^{g}\tau_{4}({\text{y}}_{i};\Psi^{\left(j\right)})} & \text{if}\sum_{i=1}^{g}\tau_{4}({\text{y}}_{i};\Psi^{\left(j\right)})y_{a_{i}}\leq 0;\\ 0 & \text{otherwise.} \end{array}\right)\\ {\hat{\mu }}_{h4}^{\left(j+1\right)}=\left\{\begin{array}{ll} \frac{\sum_{i=1}^{g}\tau_{4}({\text{y}}_{i};\Psi^{\left(j\right)})y_{h_{i}}}{\sum_{i=1}^{g}\tau_{4}({\text{y}}_{i};\Psi^{\left(j\right)})} & \text{if}\sum_{i=1}^{g}\tau_{4}({\text{y}}_{i};\Psi^{\left(j\right)})y_{h_{i}}\geq 0;\\ 0 & \text{otherwise.} \end{array}\right) \end{array}$$

The MLEs of **Σ**_*k*_, *k* = 1,…,4, at the (*j* + 1) M-step remain unchanged as in (10).

The MLEs on the (*j* + 1) iteration for (*μ*_*ak*_,*μ*_*hk*_)^*T*^, *k* = 5,…,8, are

$$\begin{array}{l} {\hat{\mu }}_{h5}^{\left(j+1\right)}=\left\{\begin{array}{ll} \frac{\sum_{i=1}^{g}\tau_{5}({\text{y}}_{i};\Psi^{\left(j\right)})y_{h_{i}}}{\sum_{i=1}^{g}\tau_{5}({\text{y}}_{i};\Psi^{\left(j\right)})} & \text{if}\sum_{i=1}^{g}\tau_{5}({\text{y}}_{i};\Psi^{\left(j\right)})y_{h_{i}}\geq 0;\\ 0 & \text{otherwise.} \end{array}\right)\\ {\hat{\mu }}_{h6}^{\left(j+1\right)}=\left\{\begin{array}{ll} \frac{\sum_{i=1}^{g}\tau_{6}({\text{y}}_{i};\Psi^{\left(j\right)})y_{h_{i}}}{\sum_{i=1}^{g}\tau_{6}({\text{y}}_{i};\Psi^{\left(j\right)})} & \text{if}\sum_{i=1}^{g}\tau_{6}({\text{y}}_{i};\Psi^{\left(j\right)})y_{h_{i}}\leq 0;\\ 0 & \text{otherwise.} \end{array}\right)\\ {\hat{\mu }}_{a7}^{\left(j+1\right)}=\left\{\begin{array}{ll} \frac{\sum_{i=1}^{g}\tau_{7}({\text{y}}_{i};\Psi^{\left(j\right)})y_{a_{i}}}{\sum_{i=1}^{g}\tau_{7}({\text{y}}_{i};\Psi^{\left(j\right)})} & \text{if}\sum_{i=1}^{g}\tau_{7}({\text{y}}_{i};\Psi^{\left(j\right)})y_{a_{i}}\geq 0;\\ 0 & \text{otherwise.} \end{array}\right)\\ {\hat{\mu }}_{a8}^{\left(j+1\right)}=\left\{\begin{array}{ll} \frac{\sum_{i=1}^{g}\tau_{8}({\text{y}}_{i};\Psi^{\left(j\right)})y_{a_{i}}}{\sum_{i=1}^{g}\tau_{8}({\text{y}}_{i};\Psi^{\left(j\right)})} & \text{if}\sum_{i=1}^{g}\tau_{8}({\text{y}}_{i};\Psi^{\left(j\right)})y_{a_{i}}\leq 0;\\ 0 & \text{otherwise.} \end{array}\right) \end{array}$$

The MLE for **Σ**_*k*_, *k* = 0,5,6,7,8, on the (*j* + 1) iteration is

$${\hat{\Sigma }}_{k}=\left(\begin{array}{cc} \frac{\sum_{i=1}^{g}\tau_{k}({\text{y}}_{i};\Psi^{\left(j\right)})y_{a_{i}}^{2}}{\sum_{i=1}^{g}\tau_{k}({\text{y}}_{i};\Psi^{\left(j\right)})} & 0\\ 0 & \frac{\sum_{i=1}^{g}\tau_{k}({\text{y}}_{i};\Psi^{\left(j\right)})y_{h_{i}}^{2}}{\sum_{i=1}^{g}\tau_{k}({\text{y}}_{i};\Psi^{\left(j\right)})} \end{array}\right).$$

#### Regularized covariance matrices in the EM algorithm

The component covariance matrices of a mixture model may be singular or near singular in the EM iterative process. When the covariance matrices corresponding to one or more components are ill-conditioned (singular or near singular), the EM algorithm breaks down. Particularly, applying the EM algorithm for a mixture model with large numbers of components when there are actually fewer groups often results in the failure of the EM algorithm due to ill-conditioning [24,25]. Indeed, this break-down of the EM algorithm may imply that clusters contain insufficient observations and too many components are used to fit the data set where there are actually fewer clusters, or clusters contain points that are of very little variation compared to other clusters. Hence, an intuitive solution to this is to decrease the number of the mixture components. However, this immediately leads to another question: how many clusters are needed. Though an active area of research, it is beyond the scope of this study. Nonetheless, various approaches have been proposed to generate numerically non-singular covariance matrices [26-33]. Among such, the regularization method proposed by Sato and Ishii [32] has been adopted to obtain numerically non-singular covariance matrices throughout this research. In Sato and Ishii [32], the regularized covariance matrix for the *k*th mixture component in the (*j* + 1) M-step is

(11) $$\Sigma_{\text{Rk}}^{\left(j+1\right)}=\Sigma_{k}^{\left(j+1\right)}+\alpha \frac{\text{tr}(\Sigma_{k}^{\left(j+1\right)})}{p}I_{p},$$

where 0 ≤ *α* ≤ 1 is a small constant and *I*_*p*_ is a *p*-dimensional identity matrix. If $\Sigma_{k}^{\left(j+1\right)}$ equals 0, then $\text{tr}(\Sigma_{k}^{\left(j+1\right)})$ is set to be a small threshold value (0.0001 in this research).

## Competing interests

The authors declare that they have no competing interests.

## Authors’ contributions

YS, LZ, AM and JO have participated in the development of the proposed model. YS has drafted the manuscript. All authors read and approved the final manuscript.
